# Dynamic Mechanical Response of Biomedical 316L Stainless Steel as Function of Strain Rate and Temperature

**DOI:** 10.1155/2011/173782

**Published:** 2011-12-20

**Authors:** Woei-Shyan Lee, Tao-Hsing Chen, Chi-Feng Lin, Wen-Zhen Luo

**Affiliations:** ^1^Department of Mechanical Engineering, National Cheng Kung University, Tainan 701, Taiwan; ^2^Department of Mechanical Engineering, National Kaohsiung University of Applied Sciences, Kaohsiung 807, Taiwan; ^3^National Center for High-Performance Computing, Hsin-Shi Tainan 744, Taiwan

## Abstract

A split Hopkinson pressure bar is used to investigate the dynamic mechanical properties of biomedical 316L stainless steel under strain rates ranging from 1 × 10^3^ s^−1^ to 5 × 10^3^ s^−1^ and temperatures between 25°C and 800°C. The results indicate that the flow stress, work-hardening rate, strain rate sensitivity, and thermal activation energy are all significantly dependent on the strain, strain rate, and temperature. For a constant temperature, the flow stress, work-hardening rate, and strain rate sensitivity increase with increasing strain rate, while the thermal activation energy decreases. Catastrophic failure occurs only for the specimens deformed at a strain rate of 5 × 10^3^ s^−1^ and temperatures of 25°C or 200°C. Scanning electron microscopy observations show that the specimens fracture in a ductile shear mode. Optical microscopy analyses reveal that the number of slip bands within the grains increases with an increasing strain rate. Moreover, a dynamic recrystallisation of the deformed microstructure is observed in the specimens tested at the highest temperature of 800°C.

## 1. Introduction

Austenitic 316L stainless steels have a range of favourable mechanical properties, including good corrosion resistance, high strength under elevated temperatures, excellent ductility, and good weldability [1, 2]. As a result, they are used for a wide variety of applications in the nuclear, chemical and aerospace industries [3–5]. In addition to their excellent mechanical properties, 316L SS alloys also have good biocompatibility and are therefore an ideal material for medical tools and surgical implants [6]. While the quasistatic mechanical properties of 316L SS have attracted significant attention [7–12], relatively little information is available regarding its dynamic mechanical behaviour under high strain rates and temperatures. Nonetheless, 316L SS components typically experience a wide range of strain rates and temperatures during their fabrication and/or service lives. Thus, to ensure the mechanical integrity of such components, it is necessary to examine the dynamic deformation and fracture behaviour of 316L SS alloy over a wide range of temperatures and strain rates.

The high strain rate mechanical properties of most engineering materials are quite different from those observed under quasi-static conditions. For example, under quasi-static loading, the fracture mechanism is dominated by ductile or brittle modes, whereas under impact loading, specimen fracture is the result primarily of the formation of adiabatic shear bands due to a localisation of the plastic flow. The formation of adiabatic shear bands has been extensively studied [13–16]. In general, the results have shown that shear band formation is sensitive to both the strain rate and the deformation temperature.

The flow stress induced within deformed materials is also dependent on the strain rate and temperature. Specifically, the flow stress increases with an increasing strain rate, but decreases with an increasing temperature. The high strain rate mechanical properties of structural materials are commonly evaluated using a split Hopkinson pressure bar (SHPB) [17, 18]. The different deformation behaviours observed under different strain rates and temperatures have been variously attributed to dislocation damping [19], thermal activation [20], dislocation generation [21], and so forth. However, the correlation between the deformation mechanism and the dynamic behaviour of 316L SS under high strain rates and temperatures has yet to be reported. Consequently, this study utilises a compressive SHPB system to investigate the dynamic mechanical behaviour of 316L SS at strain rates ranging from 1 × 10^3^ s^−1^ to 5 × 10^3^ s^−1^ and temperatures between 25°C and 800°C. The fracture mechanism of the 316L SS specimens is investigated via scanning electron microscopy (SEM). In addition, the microstructures of the impacted specimens are observed using optical microscopy (OM). Finally, the correlation between the macromechanical response of the 316L SS specimens and their microstructural evolution is examined and discussed.

## 2. Specimen Preparation and ExperimentalProcedure

**Figure 1:**
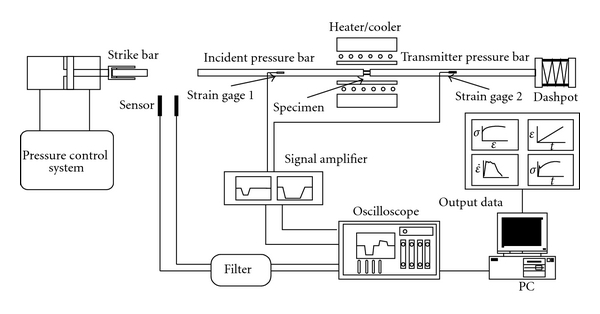
Schematic diagram of compressive split Hopkinson pressure bar system.

The impact tests were performed using commercial-grade 316L SS with the chemical composition (wt.%) shown in Table 1. The as-received material was cold drawn to a bar with a diameter of 13 mm and was then annealed at a temperature of 1000°C for 50 min in order to release the residual stress produced in the drawing process. Specimens with a length of 7 ± 0.1 mm and a diameter of 7.2 mm were machined from the bar and finished to a final diameter of 7 ± 0.1 mm via a centre-grinding process. Dynamic impact tests were conducted using an SHPB system at strain rates of 1 × 10^3^ s^−1^, 3 × 10^3^ s^−1^, and 5 × 10^3^ s^−1^, respectively, and temperatures of 25°C, 200°C, 400°C, and 800°C. In each test, the specimen was sandwiched between the incident bar and the transmitter bar of the SHPB system, and the incident bar was then impacted by a striker bar fired by a gas gun (see Figure 1).

 The test temperatures of 200°C, 400°C, and 800°C were obtained by fitting a tunnel-type electric furnace around the facing ends of the incident and transmitter bars. The elevated test temperatures induced a temperature gradient along the lengths of the two pressure bars; causing a change in both the elastic modulus of the bars and the propagation velocity of the pressure pulse. Accordingly, the original equations for the strain, strain rate, and stress were modified to the forms given by Chiddister and Malvern in [22] and the current authors in [23]. (Note that full details of the experimental procedure and analytical technique used to establish the dynamic mechanical response of the impacted specimens are presented by the current authors in [24]). 

 Specimens for microstructural analysis were prepared using standard metallographic techniques. The nucleation and growth of the adiabatic shear bands were examined using optical microscopy (Axiovert 200MAT optical microscope). Finally, the surfaces of the fractured specimens were observed using a FEI Quanta 400 F scanning electron microscope with an operating voltage of 30 kV.

## 3. Results and Discussion

### 3.1. Mechanical Behaviour and True Stress-Strain Curves

**Figure 2:**
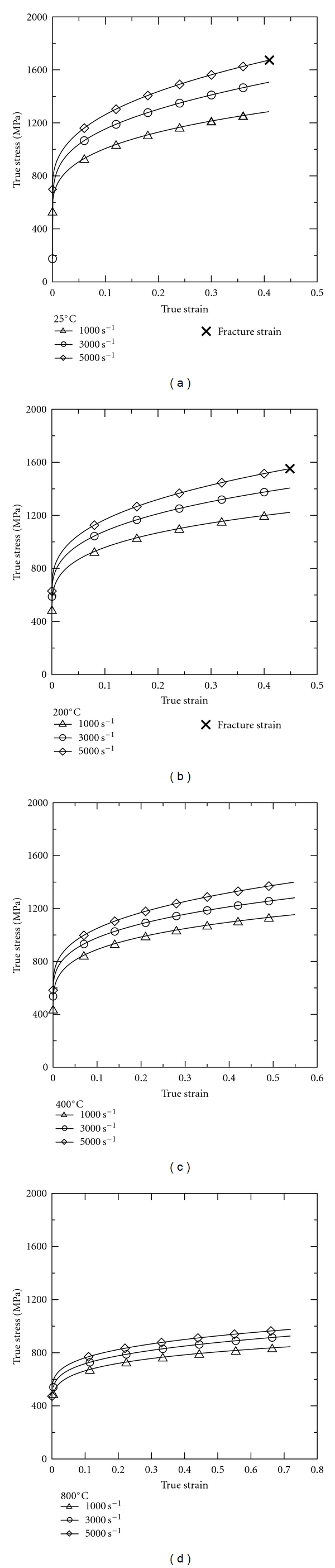
True stress-strain curves of 316L stainless steel deformed at different strain rates and temperatures of (a) 25°C; (b) 200°C; (c) 400°C; (d) 800°C.

Figures 2(a)–2(d) present the stress-strain curves of the 316L SS specimens as a function of the strain rate at deformation temperatures of 25°C, 200°C, 400°C, and 800°C, respectively. In general, the figures show that for all values of the deformation temperature, the flow stress depends on both the strain and the strain rate. Specifically, for a constant strain rate, the flow stress increases gradually with an increasing strain, while for a constant strain, the flow stress increases gradually with an increasing strain rate. It is observed that only those specimens deformed at a high strain rate of 5 × 10^3^ s^−1^ and temperatures of 25°C or 200°C fracture. In other words, it is inferred that 316L SS alloy has good ductility under low strain rate and high temperature conditions.

**Figure 3:**
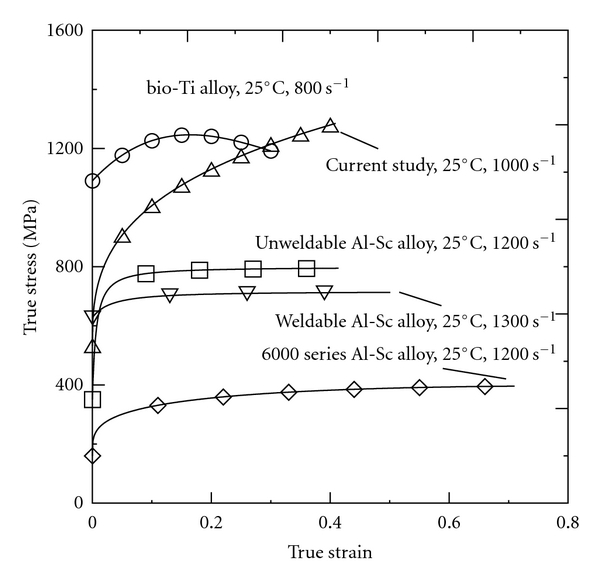
Comparison of stress-strain response of 316L stainless steel with that of biomedical Ti alloy, unweldable Al-Sc alloy, weldable Al-Sc alloy, and Al-Sc alloy, respectively.

Figure 3 compares the experimental stress-strain curve obtained for the current 316L SS alloy at a strain rate of 1 × 10^3^ s^−1^ and a temperature of 25°C with the curves presented by the present group in previous studies for biomedical Ti alloy [25], unweldable Al-Sc alloy [26], weldable Al-Sc alloy [27], and Al-Sc alloy [28] under broadly equivalent loading conditions. It can be seen that the 316L SS specimen has the highest flow stress of the five alloys at true strain of 0.4. The results also show that the mechanical properties of 316L SS are superior to those of Al-Sc alloys; indicating that Al-Sc alloy is less suitable for biomedical applications. While biomedical Ti alloy and 316L SS both have good biocompatibility and strength properties, the deformability and fracture resistance of 316L SS are superior to those of biomedical Ti alloy (see Figure 3). Furthermore, 316L SS is cheaper than biomedical Ti alloy. Thus, of the two alloys, 316L SS is better suited to biomedical applications.

**Figure 4:**
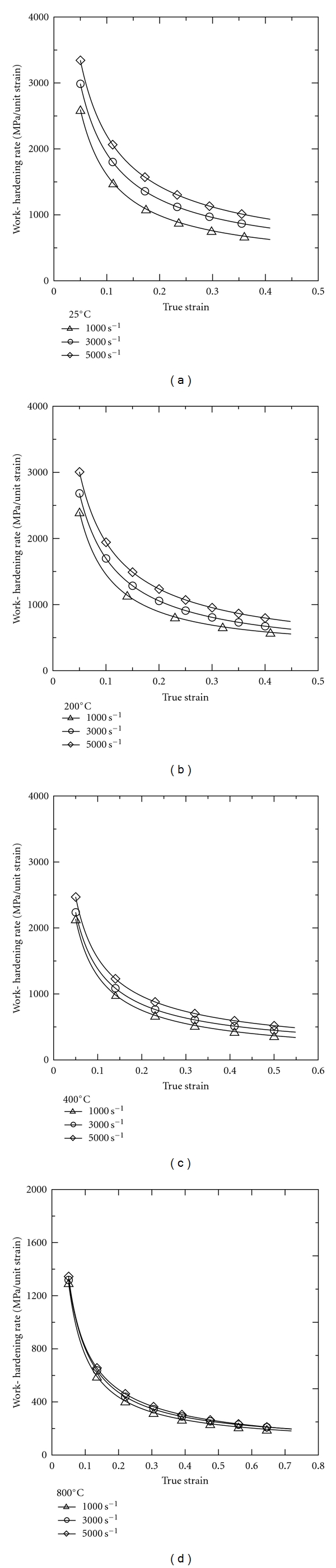
Work-hardening rate of 316L stainless steel deformed at different strain rates and temperatures of (a) 25°C; (b) 200°C; (c) 400°C; (d) 800°C.

Figures 4(a)–4(d) present the work hardening rate (∂*σ*/∂*ε*) of the 316L SS specimens as a function of the strain rate at temperatures of 25°C, 200°C, 400°C, and 800°C, respectively. The results show that for each test temperature, the work hardening rate decreases with increasing strain for a constant strain rate, but increases with increasing strain rate at a constant strain. Moreover, comparing the four figures, it is seen that the work hardening rate decreases with increasing temperature for a constant strain rate and strain. From a metallurgical viewpoint, the work-hardening rate reflects the result of a competition process between the work-hardening mechanism and the thermal-softening mechanism. In general, the work hardening effect is induced by dislocation multiplication, twin formation, and martensite transformation [29]. Due to the short timescales involved in dynamic loading, the heat generated by the plastic work done in the deformation process has insufficient time to dissipate. Consequently, a local temperature rise occurs; resulting in a thermal-softening of the deformed material [30, 31]. It is noted that the thermal-softening effect observed at high temperatures can be attributed at least in part to the suppression of martensite formation.

Table 2 indicates the work-hardening rate of the five alloys shown in Figure 3 at a true strain of 0.1, a temperature of 25°C, and a broadly similar strain rate. It can be seen that the work-hardening rate of the current 316L SS alloy is higher than that of Al-Sc alloy [26–28] or biomedical Ti alloy [25] under similar loading conditions.

### 3.2. Strain Rate Sensitivity and Thermal Activation Energy

**Figure 5:**
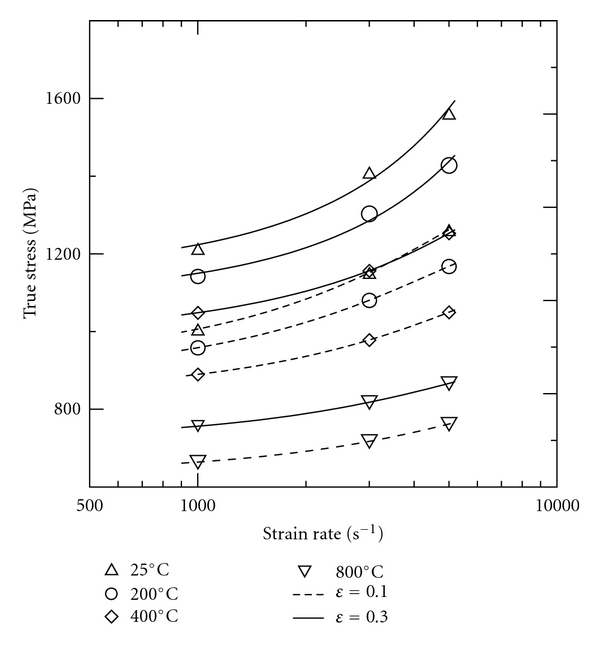
Variation of true stress with strain rate as function of temperature at true strains of 0.1 and 0.3.

**Figure 6:**
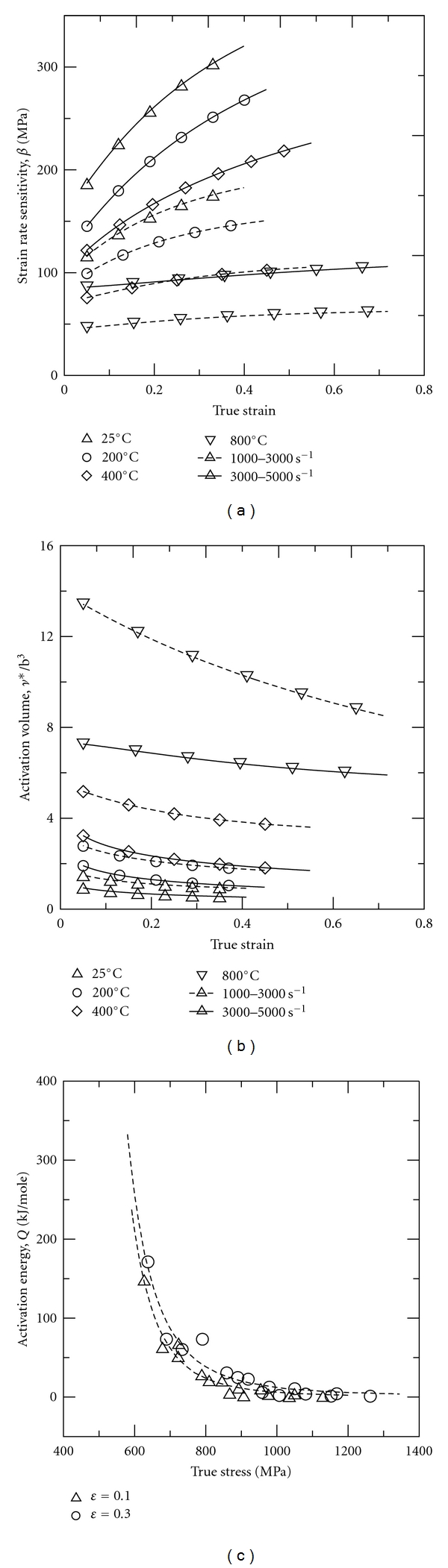
(a) Variation of strain rate sensitivity of 316L stainless steel with true strain as function of temperature and strain rate; (b) variation of activation volume of 316L stainless steel with true strain as function of temperature and strain rate; (c) variation of thermal activation energy of 316L stainless steel with true stress as function of true strain.

The stress-strain curves presented in Figures 2(a)–2(d) indicate that the mechanical behaviour of 316L SS alloy is significantly dependent on the strain rate. The strain rate dependence of the 316L SS specimens can be investigated by plotting the flow stress against the logarithmic strain rate at a constant strain.  Figure 5 shows the variation of the flow stress with the logarithmic strain rate as a function of the temperature at true strains of 0.1 and 0.3, respectively. It is seen that the flow stress increases dramatically with both an increasing strain rate and an increasing strain. The higher flow stress at a higher strain rate suggests that the dynamic deformation behaviour of 316L SS is governed by different rate-controlling mechanisms in different strain rate ranges. In general, the strain rate effect at a given temperature can be quantified via the following strain rate sensitivity parameter [32]:

(1) $$\beta =\left(\frac{\partial \sigma }{\partial \ln\dot{\varepsilon }}\right)=\frac{\sigma_{2}-\sigma_{1}}{\ln\left({\dot{\varepsilon }}_{2}/{\dot{\varepsilon }}_{1}\right)},$$

where the flow stresses *σ*_2_ and *σ*_1_ are obtained from impact tests conducted at average strain rates of ${\dot{\varepsilon }}_{2}$ and ${\dot{\varepsilon }}_{1}$, respectively, and are calculated at the same value of the plastic strain.  Figure 6(a) shows the variation of the strain rate sensitivity of the present 316L SS specimens with the true strain at different temperatures and strain rates. The results show that the strain rate sensitivity increases with increasing strain and strain rate, but decreases with increasing temperature.  Table 3 indicates the strain rate sensitivity of the various alloys shown in Figure 3 at a true strain of 0.1 and a temperature of 25°C. It is found that for a strain rate range of 1000–3000 s^−1^, the present 316L SS alloy is more sensitive to the strain rate than the Al-Sc alloys [27, 28], but is less sensitive to the strain rate than the biomedical Ti alloy [25]. 

In general, the strain rate sensitivity index of a material represents the extent to which the flow stress is affected by the strain rate. The lower strain rate sensitivity of Al-Sc alloys compared to 316L SS and biomedical Ti alloy implies that such alloys are insufficiently strong and durable to withstand high physiological loads during their service lives. As a result, they are unsuitable for biomedical applications.  Table 3 shows that the strain rate sensitivity of biomedical Ti alloy is significantly higher than that of 316L SS. In other words, the flow stress in biomedical Ti alloy increases more rapidly than that in 316L SS under dynamic loading conditions. Consequently, a strength mismatch occurs between artificial implants fabricated from biomedical Ti alloys and the surrounding natural structure. Overall, the results presented in Table 3 suggest that 316L SS is a more suitable material for biomedical applications due to its relatively lower strain rate sensitivity, which leads in turn to a lower strength mismatch between the implant and the surrounding structure. As a result, 316L SS is ideally suited for such biomedical applications as bone, acetabula cup (one half of an artificial hip joint), and knee cap replacements, as well as for screws, plates, and prostheses in odontology and orthopaedic applications.

The plastic deformation of 316L SS is a thermally activated process and can be expressed in the form of the following Arrhenius equation [33]:

(2) $$\dot{\varepsilon }={\dot{\varepsilon }}_{0}\exp\left[-\frac{\Delta G^{*}}{K_{\text{b}}T}\right],$$

where *ε*_0_ is the frequency factor, ΔG* is the activation energy, *K*_b_ is the Boltzmann constant, and *T* is the absolute temperature. According to [34, 35], ΔG*can be derived as

(3) $$\Delta G^{*}=-T\nu^{*}{\left[\frac{\partial \sigma }{\partial T}\right]}_{\dot{\varepsilon },\varepsilon },$$

where ν* is the activation volume and can be obtained as [36]

(4) $$\nu^{*}=kT\left(\frac{\partial \ln\dot{\varepsilon }}{\partial \sigma }\right)=\frac{kT}{\beta }.$$

Figure 6(b) presents the results obtained for the activation volume of the present 316L SS specimens by substituting the stress-strain data in Figures 2(a)–2(d) into (4). (Note that the activation volume data are normalised by b^3^, where b is the Burger's vector and has a value of 2.58 Å for 316L SS). It can be seen that for a constant strain and strain rate, the activation volume increases with increasing temperature. However, for a constant temperature, the activation volume decreases with increasing strain rate.  Table 4 compares the activation volume of the present 316L SS alloy with that of the Al-Sc alloys tested in [26–28] and the biomedical Ti alloy tested in [25]. Note that in every case, the true strain is equal to 0.1 and the temperature is equal to 25°C. It is seen that the activation volume of 316L SS is higher than that of the biomedical Ti alloy, but lower than that of the Al-Sc alloys. 

Figure 6(c) plots the variation of the activation energy with the flow stress at true strains of 0.1 and 0.3, respectively. The results show that the flow stress increases as the activation energy decreases. In other words, as the activation energy reduces, the ability of the mobile dislocations in the deformed microstructure to overcome short-range barriers also reduces, and thus the flow stress increases.  Table 5 compares the maximum activation energy of the current 316L SS alloy with that of unweldable Al-Sc alloy [37] and biomedical Ti alloy [25]. It is seen that the maximum activation energy of the 316L SS alloy is slightly lower than that of the biomedical alloy, but is significantly higher than that of the unweldable Al-Sc alloy.

### 3.3. Temperature Effect

**Figure 7:**
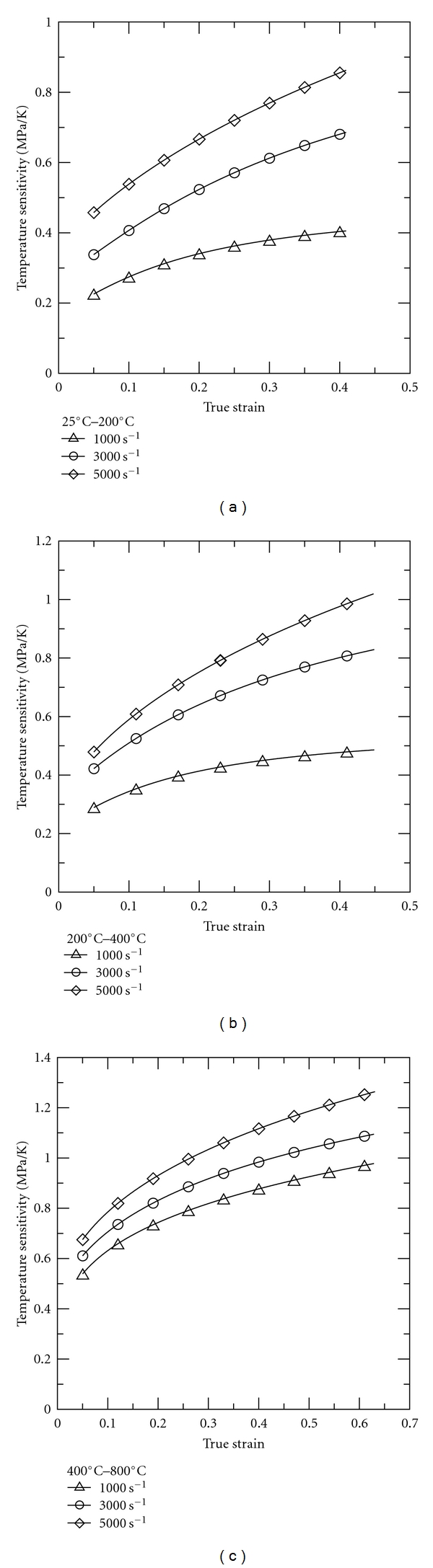
Temperature sensitivity of 316L stainless steel at temperatures in the range of (a) 25°C to 200°C; (b) 200°C to 400°C; (c) 400°C to 800°C.

In practice, it is virtually impossible to measure the temperature rise (Δ*T*) induced during high strain rate loading using direct experimental methods. As a result, Δ*T* is generally estimated using the integral equation  Δ*T* = 1/(*ρC*_*p*_)∫_0_^*ε*^ *σdε* [38], where *ρ* is the density (8.0 g/cm^3^), *Cp* is the heat capacity (500 J/(kg*·*K)), *σ* is the stress, and *dε* is the strain interval.  Table 6 presents the variation of Δ*T* with the true strain as a function of the strain rate and temperature. It is observed that Δ*T* increases with increasing strain rate and strain, but decreases with increasing temperature. The thermal-softening effect caused by the local temperature rise can be quantified via the following temperature sensitivity parameter [39]:

(5) $$n_{a}\;=\left|\frac{\left(\sigma_{2}-\sigma_{1}\right)}{{\left(T_{2}-T_{1}\right)}_{\dot{\varepsilon }}}\right|,$$

where the stresses *σ*_2_ and *σ*_1_ are obtained from tests conducted at temperatures *T*_2_ and *T*_1_, respectively. As shown in Figures 7(a)–7(c), the temperature sensitivity of the 316L SS specimens increases significantly with increasing temperature. Moreover, for a constant temperature and true strain, the temperature sensitivity increases with increasing strain rate. Thus, it can be inferred that the strain rate-induced strengthening effect is restrained by a thermal-softening effect. This restraining effect is particularly pronounced at higher strain rates (5 × 10^3^ s^−1^) and higher temperatures (800°C).

### 3.4. Fracture Surface Observations and Microstructural Evolution

**Figure 8:**
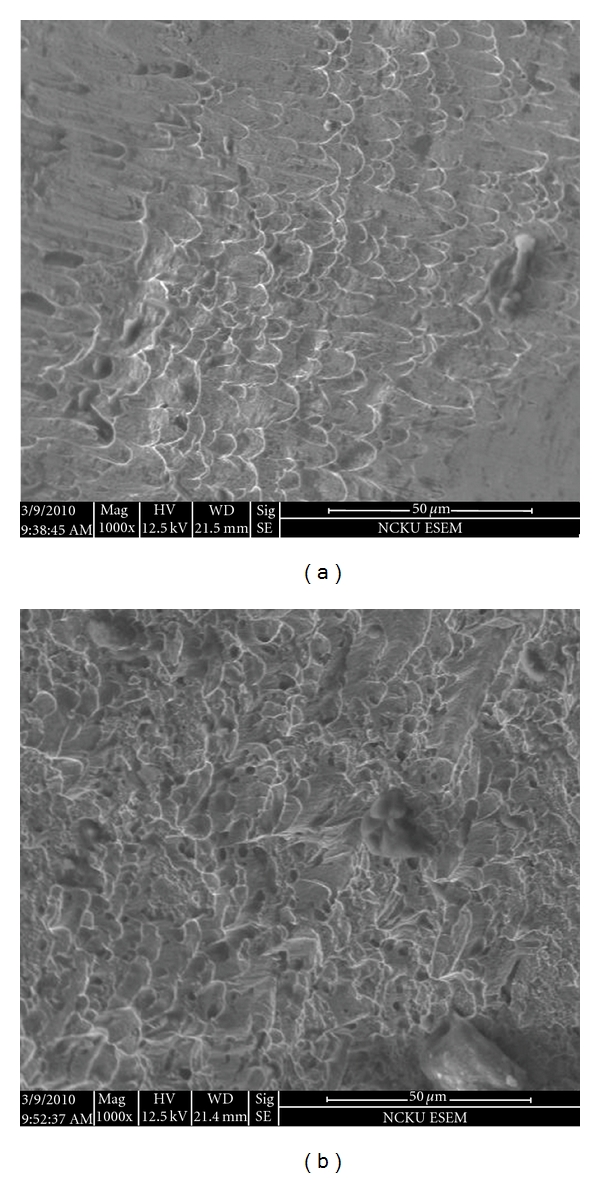
SEM fractographic images of 316L stainless steel specimens deformed at strain rate of 5 × 10^3^ s^−1^ and temperatures of (a) 25°C and (b) 200°C.

In the impact tests performed in this study, only those specimens deformed at a strain rate of 5 × 10^3^ s^−1^ and temperatures of 25°C or 200°C suffered catastrophic failure. Figures 8(a) and 8(b) present SEM micrographs of the specimens deformed at 25°C and 200°C, respectively. Both fracture surfaces are characterised by a transgranular dimple-like structure. Thus, it is inferred that both specimens fracture in a predominantly ductile mode as a result of localised shearing. Comparing the two figures, it is seen that the density of the dimples increases with increasing temperature. In other words, the ductility of 316L SS increases at higher temperatures.

**Figure 9:**
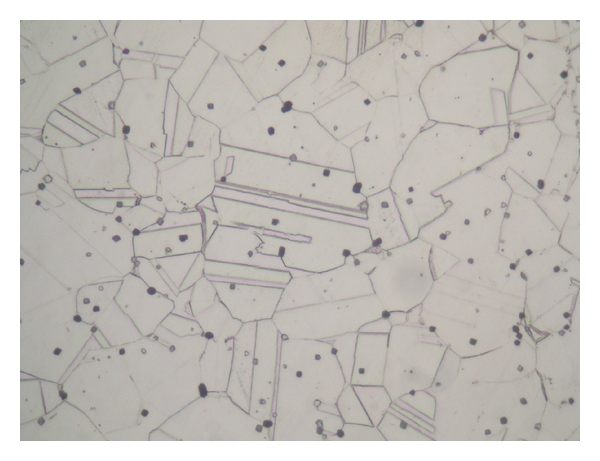
Optical micrograph of undeformed 316L stainless steel specimen.

**Figure 10:**
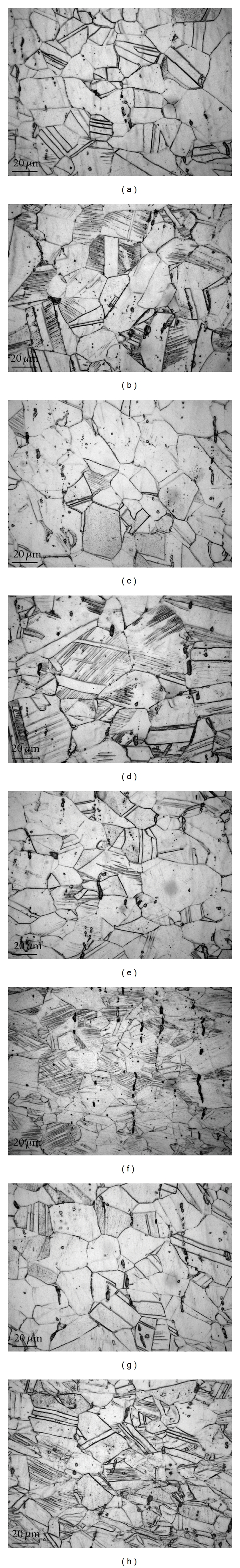
Metallographic images of 316L stainless steel specimens deformed at (a) 25°C and 1 × 10^3^ s^−1^; (b) 25°C and 5 × 10^3^ s^−1^; (c) 200°C and 1 × 10^3^ s^−1^; (d) 200°C and 5 × 10^3^ s^−1^; (e) 400°C and 1 × 10^3^ s^−1^; (f) 400°C and 5 × 10^3^ s^−1^; (g) 800°C and 1 × 10^3^ s^−1^; (h) 800°C and 5 × 10^3^ s^−1^.

Figure 9 presents an optical micrograph of an undeformed 316L SS specimen. A small number of annealing twins are observed within the grains, which suggests that the microstructure has a FCC structure. Figures 10(a) and 10(b) present optical micrographs of the 316L SS specimens deformed at a temperature of 25°C and strain rates of 1 × 10^3^ s^−1^ and 5 × 10^3^ s^−1^, respectively.  Figure 10(a) shows that high strain rate loading prompts the formation of slip bands within the grains. It is noted that the slip bands within each grain are straight and parallel, but are oriented at a different angle from those in the neighboring grains. Observing Figure 10(b), it is seen that the number of slip bands increases as the strain rate is increased. A similar tendency is observed in the specimens deformed at higher temperatures of 200°C, 400°C, and 800°C, respectively (see Figures 10(c)–10(h)). According to Fujita et al. [40] and Sakai [41], dynamic recrystallisation (or some other form of recovery mechanism) commonly occurs under high temperature deformation conditions. In the present impact tests, the highest deformation temperature (800°C) is more than 50% of the melting point of 316L SS (1380°C). Thus, as shown in Figure 10(h), the microstructure of the specimen deformed at a strain rate of 5 × 10^3^ s^−1^ and a temperature of 800°C shows signs of dynamic recrystallisation in some regions.

 In general, the present results show that the flow stress in 316L SS increases with increasing strain rate, but decreases with increasing temperature. Moreover, the microstructural observations show that the number of slip bands within the individual grains increases as the strain rate is increased or the temperature is decreased. The slip bands act as obstacles to dislocation motion and enhance the interaction of the dislocations with the surrounding slip bands. Thus, the resistance encountered by the moving dislocations increases at higher strain rates and lower temperatures. This accounts for the strengthening effect observed at higher strain rates and lower temperatures in Figure 2.

## 4. Conclusions

This study has examined the dynamic mechanical properties of biomedical 316L SS under strain rates ranging from 1 × 10^3^ s^−1^ to 5 × 10^3^ s^−1^ and temperatures of 25°C to 800°C. In general, the results have shown that the dynamic mechanical behaviour and microstructural evolution of 316L SS are significantly dependent on both the strain rate and the temperature. The stress-strain curves have shown that the flow stress and work-hardening rate increase with increasing strain rate, but decrease with increasing temperature. The strain rate sensitivity decreases with increasing temperature. Conversely, the thermal activation energy increases with increasing temperature, but decreases with increasing flow stress. Under high strain rate loading conditions, a notable deformation-induced temperature rise occurs, resulting in a thermal softening effect. Of all the specimens, only those deformed at a strain rate of 5 × 10^3^ s^−1^ and temperatures of 25°C or 200°C suffer catastrophic failure. Thus, it is inferred that 316L SS alloy has good ductility under low strain rate and high temperature loading conditions. SEM observations have shown that the fracture surfaces are characterised by a transgranular dimple-like structure. In other words, the specimens fail in a predominantly ductile mode. Finally, the microstructural observations have shown that a dynamic recrystallisation of the deformed microstructure occurs in the specimens tested under the highest deformation temperature of 800°C.

## Figures and Tables

**Table 1:** Chemical composition of as-received 316L stainless steel.

Element	wt.%	Element	wt.%
C	0.02	Ni	10.09
Si	0.46	Cr	16.76
Mn	1.77	Mo	2.02
P	0.029	N	0.06
S	0.027	Fe	Balance

**Table 2:** Work-hardening rate of various alloys at 25°C.

Metal	Strain rate (s^−1^)	True strain	Work-hardening rate (MPa/unit strain)	Reference
316L stainless steel	1000	0.1	1500	current study
6000 series Al-Sc alloy	1200	0.1	403.2	[28]
Unweldable Al-Sc alloy	1200	0.1	200	[26]
Weldable Al-Sc alloy	1300	0.1	160	[27]
Biomedical Ti alloy	800	0.1	250	[25]

**Table 3:** Strain rate sensitivity of various alloys at 25°C.

Metal	Strain rate (s^−1^)	True strain	Strain rate sensitivity *β* (MPa)	Reference
316L stainless steel	1000–3000	0.1	130	current study
6000 series Al-Sc alloy	0.001–3200	0.1	4	[28]
Unweldable Al-Sc alloy	1200–3200	0.1	66.8	[26]
Weldable Al-Sc alloy	1300–3200	0.1	58.6	[27]
Biomedical Ti alloy	800–3000	0.1	203	[25]

**Table 4:** Activation volume of various alloys at 25°C.

Metal	Strain rate (s^−1^)	True strain	Activation volume (ν*/b^3^)	Reference
316L stainless steel	1000–3000	0.1	1.3	current study
6000 series Al-Sc alloy	1200–3200	0.1	4.2	[28]
Unweldable Al-Sc alloy	1200–3200	0.1	1.9	[26]
Weldable Al-Sc alloy	1300–3200	0.1	2.4	[27]
Biomedical Ti alloy	800–3000	0.1	1.11	[25]

**Table 5:** Activation energy of various alloys at 25°C.

Metal	Max. activation energy (kJ/mole)	Corresponding flow stess (MPa)	Reference
316L stainless steel	150	640	current study
Unweldable Al-Sc alloy	48	400	[37]
Biomedical Ti alloy	160	703	[25]

**Table 6:** Variation of deformation-induced temperature rise as function of temperature and strain rate.

*T* (°C)	Strain rate (s^−1^)	Temperature rise ΔT (K)				
		*ε* = 0.05	*ε* = 0.1	*ε* = 0.2	*ε* = 0.3	*ε* = 0.4
25	5000	12.83	27.85	61.76	99.33	139.72
	3000	12.48	27.06	59.80	85.3	121.2
	1000	10.29	22.31	53.2	79.4	100.3

200	5000	11.94	25.86	57.12	91.54	128.37
	3000	11.13	24.05	52.85	82.36	109
	1000	9.85	21.32	50.2	73.4	95.6

400	5000	10.96	23.54	51.41	81.76	113.95
	3000	10.26	22.00	47.91	75.97	99.4
	1000	9.22	19.89	39.3	65.9	89.6

800	5000	8.38	17.62	37.52	58.68	80.78
	3000	7.81	16.49	35.25	55.23	76.10
	1000	7.15	15.19	34.2	51.3	72.1
